# Relationship between the Functional Oral Intake Scale (FOIS) and the Self-Efficacy Scale among Cancer Patients: A Cross-Sectional Study

**DOI:** 10.3390/healthcare8030269

**Published:** 2020-08-13

**Authors:** Yuhei Matsuda, Masaaki Karino, Takahiro Kanno

**Affiliations:** Department of Oral and Maxillofacial Surgery, Shimane University Faculty of Medicine, Shimane 693-8501, Japan; karino71@med.shimane-u.ac.jp (M.K.); tkanno@med.shimane-u.ac.jp (T.K.)

**Keywords:** functional oral intake scale (FOIS), self-efficacy scale for advanced cancer (SEAC), oral health-related self-efficacy scale for patients with cancer (OSEC), cancer, dysphagia, nutrition

## Abstract

A few studies have provided detailed reports suggesting that subjective swallowing disorders may be related to dysphagia. Therefore, we verified the relationship between oral health-related self-efficacy and dysphagia severity in cancer treatment using a cross-sectional study. Participants included patients undergoing treatment for cancer at Shimane University Hospital in Shimane, Japan, and those receiving outpatient treatment at the hospital’s Oral Care Center between August 2018 and April 2019. In all, 203 participants enrolled in the study and completed the Functional Oral Intake Scale (FOIS), the Self-efficacy Scale for Advanced Cancer (SEAC), and the Oral Health-related Self-Efficacy Scale for Patients with Cancer (OSEC). Multivariate analysis showed a statistically significant correlation between the low FOIS score and the SEAC subscales of Activities of Daily Living Self-efficacy (ADE) (odds ratio 1.04, 95% [CI] 1.00–1.07) and Symptom Coping Self-efficacy (SCE) (odds ratio 0.61, 95% [CI] 0.42–0.88). Based on the Jonckheere-Terpstra test, the SEAC and the OSEC tended to increase as the category of the FOIS progressed. To conclude, self-efficacy played an important role in dysphagia and may affect the severity of dysphagia in cancer patients.

## 1. Introduction

In cancer patients, it is highly likely that symptoms such as swallowing dysfunction (dysphagia) and appetite loss will become a focus of supportive care [1]. Dysphagia may lead to greater distress for not only the cancer patients (due to the withholding or discontinuation of consumption of fluids and solids) but also for their caregiver(s) (due to the increased physical and mental burden of preparing and assisting the patient with meals) [2]. In cancer patients, many factors can cause dysphagia. It can significantly impact physical and psychological wellbeing, and its management can become complex and multifactorial [3]. There are numerous reports of dysphagia and appetite loss in cancer patients (stomach, colorectal, lung, breast, and head/neck) [4,5,6,7,8]. Moreover, Jacqui et al. reported the proportion of patients with 14 types of cancers who reported dysphagic symptoms as follows: any dysphagia (54%), dysphagia for liquids (20%), and dysphagia for solids (46%). Significantly more head and neck cancer (HNC) patients and significantly fewer breast cancer patients reported dysphagia; however, there were no differences between other tumor types [7]. Additionally, dysphagia is associated with various symptoms: taste changes, xerostomia, voice changes, smell changes, thick mucus, masticatory disorders, mouth/throat pain, and trismus [7].

Dysphagia, in general, can be caused by either (1) severe neurological impairment (e.g., stroke, myasthenia gravis, inflammatory myopathies, Parkinson’s disease, and amyotrophic lateral sclerosis), (2) structural damage (e.g., trauma caused by the intubation or treatment of malignancies, especially HNC), (3) medication or toxic/drug side-effects, (4) presbyphagia, or (5) phagophobia [9,10,11]. Dysphagia can not only lead to malnutrition, weight loss, and aspiration pneumonia, but also affect health-related quality of life (HRQoL), including psychological and social aspects [12]. Youssof et al. reported that dysphagia severity had stronger associations with mental, rather than physical, components of HRQoL in patients with oculopharyngeal muscular dystrophy [13]. The literature suggests that dysphagia may affect an individual on a psychological level, where risks of suffocation, severe coughing, and vomiting may increase anxiety and lower self-esteem [14]. From a social perspective, mealtimes may be very stressful, and visiting a restaurant may no longer be pleasant [15,16]. Furthermore, patients with dysphagia can become isolated, feel excluded by others, and experience anxiety and distress at mealtime [17].

On the other hand, dysphagia severity may not be determined by constitutional and functional factors alone. In stroke and laryngectomy patients with dysphagia, psychological aspects such as self-efficacy play an important role in rehabilitation and eating habits [18,19]. In other words, what the patients with dysphagia can or cannot eat is affected by self-efficacy of deglutition. In other words, we, as medical and dental practitioners, should pay attention to the gap between the foods that cancer patients can subjectively or objectively eat.

Bandura proposed that self-efficacy is an individual’s expectation of the extent to which she/he can implement the specific actions needed to produce a specific result [20]. People’s self-efficacy beliefs determine their emotions, thoughts, behaviors, and motives. In clinical practice, self-efficacy refers to the patients’ perceptions of their abilities to take actions needed to improve and maintain health, such as control weight, engage in physical activity, and control alcohol use [21,22,23]. In a prospective cohort study of 100 patients with either transient ischemic attack or ischemic stroke, Brouwer et al. found that a baseline of self-efficacy, as determined by the patient’s responses on the questionnaires, was the strongest predictor of a patient’s intention to adopt a healthy diet (95% CI, 0.23–0.75) [24].

Self-efficacy can be evaluated by several scales such as the General Self-Efficacy Scale (GSES) and the Self-Efficacy for Advanced Cancer (SEAC). The SEAC was described by Hirai et al. as a scale designed specifically for oncology. The SEAC comprises 18 items under three factors (affect regulation self-efficacy, symptom coping self-efficacy, and activities of daily life self-efficacy) [25]. We have used the Oral Health-related Self-efficacy Scale for Cancer Patients (OSEC) in a previous study [26]. The OSEC is a 17-item scale comprising five subscales: Oral Function Self-efficacy (OFE; four items), Dental Visit Self-efficacy (DVE; three items), Adverse Effects Self-efficacy (AEE; four items), Symptom Coping Self-efficacy (SCE; three items), and Brushing Habits Self-efficacy (BHE; three items). A randomized controlled trial by Gillham and Endacott showed the self-efficacy of enhanced prevention, which consisted of providing patients with additional counseling, motivational interviewing, and frequent telephone follow-ups after suffering a minor stroke [27]. Of the 52 patients enrolled in the trial, half received this intervention, while the control group received “conventional care.” On average, patients in the “enhanced secondary prevention group” increased their consumption of fruits and vegetables by 7.6 servings per week, while the patients in the control group only increased their consumption by 2.0 servings per week (*p* = 0.03), indicating that enhancing self-efficacy can significantly impact the stroke patients’ dietary choices [27].

However, only a few studies provided detailed reports showing that the lack of self-efficacy can lead to dysphagia in cancer patients. Therefore, we verified the relationship between self-efficacy and dysphagia severity in cancer treatment using a cross-sectional study.

## 2. Materials and Methods

This study used the same data set as the previous study. However, the purpose and statistical analysis are different from the previous study [26].

### 2.1. Participants

This study included participants from the population of cancer patients being treated at the Shimane University Hospital in Shimane, Japan, and those who received outpatient treatment at the hospital’s Oral Care Center. The inclusion criteria were: (1) treated for cancer at Shimane University Hospital, (2) outpatient treatment at the Oral Care Center, Shimane University Hospital, (3) aged 20 years or older, and (4) ability to complete the self-administered written questionnaire. The exclusion criteria were: (1) currently being treated for a mental disorder and (2) having a history of a mental disorder.

The data were collected from August 2018 to April 2019. Participants were recruited using a sequential sampling method.

### 2.2. Ethical Considerations

The medical ethics committee of Shimane University Faculty of Medicine approved this study (approval number 3243). Written informed consent was obtained from all individual participants included in the study. Altogether, 203 participants enrolled in the study.

### 2.3. Measurements

The surveyed items were as follows: patient characteristics (age, gender, body mass index (BMI), alcohol consumption, Brinkman index, number of co-residents, employment), underlying characteristics of solid cancer (primary tumor site, cancer stage, treatment type, number of months since last treatment, Eastern Cooperative Oncology Group Performance Status), and intraoral findings (number of teeth, dentures, brushing times per day, family dentist, dental visit(s) in the 12 months prior to the test day).

#### 2.3.1. Functional Oral Intake Scale (FOIS)

Oral intake and nutritional status were assessed using the FOIS. Scores ranged from one to seven, with higher scores indicating better swallowing function (Table 1) [28].

#### 2.3.2. Self-Efficacy Scale for Advanced Cancer (SEAC)

The SEAC is an 18-item scale comprising three subscales: Symptom Coping Self-efficacy (SCE), Activities of Daily Living Self-efficacy (ADE), and Affect Regulation Self-efficacy (ARE). Each subscale includes six items on an 11-point response scale, ranging from zero (not at all confident) to 5 (50% confident) to 10 (totally confident). The final subscale scores were calculated by summing the scores of each subscale.

#### 2.3.3. Oral Health-Related Self-Efficacy Scale for Patients with Cancer (OSEC)

The OSEC is a 17-item scale comprising five subscales: Oral Function Self-efficacy (OFE) (four items), Dental Visit Self-efficacy (DVE) (three items), Adverse Effects Self-efficacy (AEE) (four items), Symptom Coping Self-efficacy (SCE) (three items), and Brushing Habits Self-efficacy (BHE) (three items). Response options are on a four-point Likert scale from 1 (not at all confident) to 4 (totally confident). The scores on the individual items within subscales were summed to obtain total subscale scores [26].

### 2.4. Statistical Analysis

All statistical analyses were performed using SPSS (ver. 26; SPSS Japan Inc., Tokyo, Japan). We calculated two-tailed p-values in all the analyses. The alpha level of significance was set at 0.05. The participants’ characteristics were analyzed using descriptive statistics.

The grade of FOIS was categorized as level 1 to 6 (Low FOIS) or level 7 (High FOIS) based on the presence or absence of restrictions. For the inferential analysis, the Chi-squared test was used to compare proportions. For the non-parametric quantitative determinations of two groups, the Mann–Whitney U test was used. The correlation of each variable with the grade of FOIS was tested by stepwise multivariate logistic regression analysis.

Additionally, as a subgroup analysis, the grade of FOIS was categorized as level 1 to 3 (poor FOIS: tube feeding), levels 4 and 5 (moderate FOIS: total oral diet requiring special preparation) or levels 6 and 7 (good FOIS: total oral diet without special preparation). For verifying the stepwise increase or decrease in correlation between FOIS and SEAC or OSEC’s different subscales, the Jonckheere–Terpstra test was used as a trend test.

## 3. Results

### 3.1. Participants’ Characteristics

Table 2 presents the characteristics of the participants (*n* = 203). The median age was 71 years (male: 63.5%, female: 36.5%). The median BMI was 21.7. Alcohol consumption per week was 0 days (range: 0–7) and the Brinkman Index was 0 (range: 0–4480). The median number of co-residents was 2 (0–7). About 72 (35.5%) and 131 (64.5%) patients were employed and unemployed, respectively.

### 3.2. Comparison of High and Low FOIS Groups

Table 3 summarizes the variables measured in the high FOIS and low FOIS groups. Statistically significant differences were found in age, gender, BMI, Brinkman index, primary tumor site (lung, breast, and head/neck), cancer stage, treatment type (surgery, surgery plus chemotherapy, surgery plus chemotherapy plus radiotherapy), performance status, number of teeth, brushing times, family dentist, dental visit(s), SEAC (ARE, ADE, and total score), and OEAC (OFE and total score). No significant differences were found in other variables between the two groups.

### 3.3. Multivariate Analysis

Multivariate analysis showed a statistically significant correlation between the low FOIS score and number of co-residents (odds ratio 0.70, 95% confidence interval [CI] 0.53–0.92), stomach cancer (odds ratio 5.05, 95% [CI] 1.01–25.33), colorectal cancer (odds ratio 42.74, 95% [CI] 5.71–319.92), liver cancer (odds ratio 46.95, 95% [CI] 3.77–584.4), lung cancer (odds ratio 37.13, 95% [CI] 7.62–181.1), prostate cancer (odds ratio 11.2, 95% [CI] 1.69–74.29), breast cancer (odds ratio 334.15, 95% [CI] 6.21–17969.17), cancer stage (odds ratio 0.60, 95% [CI] 0.38–0.94), Eastern Cooperative Oncology Group Performance Status (odds ratio 0.52, 95% [CI] 0.31–0.90), number of teeth (odds ratio 1.16, 95% [CI] 1.09–1.23), family dentist (odds ratio 6.55, 95% [CI] 2.07–20.75), the ADE of SEAC (odds ratio 1.04, 95% [CI] 1.00–1.07), and the SCE of SEAC (odds ratio 0.61, 95% [CI] 0.42–0.88) (Table 4).

### 3.4. Sub Group Analysis (Trend Test)

**Figure 1:**
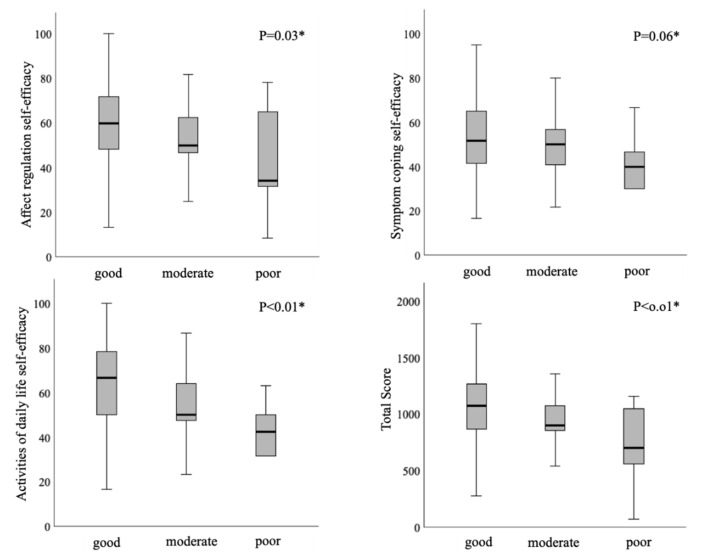
Stepwise correlation between the Self-efficacy Scale for Advanced Cancer (SEAC) and the Functional Oral Intake Scale (FOIS) using the Jonckheere–Terpstra test.

**Figure 2:**
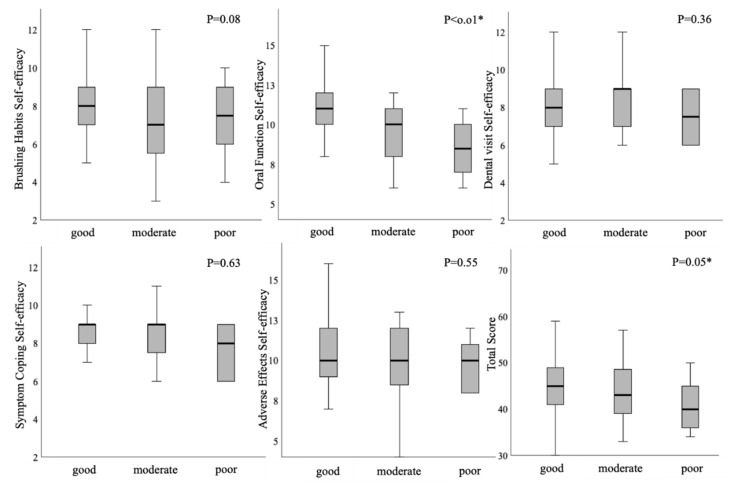
Stepwise correlation between the Oral Health-related Self-Efficacy Scale for Patients with Cancer (OSEC) and the Functional Oral Intake Scale (FOIS) using the Jonckheere–Terpstra test.

The stepwise increase was observed in each subscale of the SEAC (ARE, SCE, ADE, and total score) and the OSEC (OFE and total score) with the progression of the FOIS category using the Jonckheere–Terpstra test (Figure 1 and Figure 2). As the category of the FOIS progressed, the SEAC and the OSEC tended to increase.

## 4. Discussion

### 4.1. Generalizability from the Demographic Data

This study represents several factors of self-efficacy that affect the swallowing function. The prevalence of malnutrition in Europe and North America is 1–15% among non-institutionalized older adults, 25–60% among older adults in geriatric care facilities, 35–65% among older adults in hospitals, and a similar level in our sample (43.3%) [29]. Additionally, Jacqui et al. reported similar characteristics (male: 49%; mean age: 59; tumor type: 20.5% hematology, 18.4% breast, 11.3% HNC, 10% gynecology, 8.8% upper gastrointestinal, 8.8% colorectal, 8.4% skin/melanoma, 4.6% bone soft tissue, 3.8% lung, 5.4% other; patient setting: 21.3% inpatient, 56.1% chemotherapy, 22.6% radiotherapy) and prevalence of patient-reported dysphagia (54%) [7]. However, most of our sample had solid cancer and the most common treatment type was surgery, which may have led to the low prevalence of dysphagia as compared with the previous study. In regards to oral health status, previous studies reported that denture use was 15.9% (our study result: 57.4%), and prevalence of a dental visit(s) over the last 12 months was 52.1% (our study result: 41.1%) [28,29]. In our study, the number of teeth was less than other studies, which may affect the OSEC and the FOIS scores. Globally, we may be the first to report the FOIS score of general cancer patients; however, the tendency that the HNC was treated by ablative radical surgery with/without reconstructive surgery caused the FOIS score to deteriorate, which was consistent with previous reports [30,31]. The study by Hirai et al. on self-efficacy (SEAC) in advanced cancer patients showed similar results (ARE: 57.7–84.8, SCE: 53.6–61.2, ADE: 64.8–72.9). Thus, the generalizability of this study was limited to early-stage cancer patients who were treated by initial treatment.

### 4.2. Comparison of the High and Low FOIS Groups by Swallowing Function

The factors influencing swallowing functions were age, sex, BMI > 30, smoking status, alcohol use, cognitive factor (depression, anxiety, and psychological distress), dental status, eating habits (loss of appetite, mouth pain, complaints about the taste of the food and ability to eat independently), medical factors (vision or hearing problems, neurological disorders, weight loss, frailty and number of medications (last 7 days), physical limitations and cancer]) and social factors (highest level of education, income satisfaction, type of housing, number of cohabitants, and perceived satisfaction with social support) [32,33,34,35,36,37]. The factors reported in this study have also been reported by previous studies; thus, our result from comparing the high and low FOIS groups was not contradicted. Notably, there were significant differences between the high and low FOIS groups on SEAC (ARE, ADE, and total score) and OSEC (OFE and total score). These self-efficacy-related factors may be the new factors influencing swallowing functions. The self-efficacy expectations are positively and significantly associated with the initiation and maintenance of healthy behaviors [38,39]. It is not clear or at least not clinically convincing that the patients themselves should decide by themselves based on the self-efficacy, but it can be a reasonable guideline for performing and making selections of food to eat. Thus, self-efficacy may decide the ability of cancer patients to eat appropriate food or select the food. Moreover, these abilities may influence the probability of weight loss, appetite loss, and recurrence of cancer [40]. In this study, a multivariate analysis showed the factors of the FOIS (number of co-residents, cancer type and stage, performance status, number of teeth, family dentist, ADE and SCE of SEAC). On the other hand, the factor of the OSEC did not associate with the swallowing function against our expectations. According to Bandura, the development of self-efficacy is thought to be based upon past successful experiences with the specific behavior [20]. Therefore, patients who experience these swallowing disorders after the deterioration of daily activities may have difficulty drawing on past successful experiences, thus reducing their confidence in performing the swallowing function. In other words, this may interact with the OSEC.

### 4.3. Tendency of the FOIS and Self-Efficacy

In sub-group analysis, all subscales of the SEAC, the OFE and the total score of the OSEC showed a stepwise increase in the FOIS score. Thus, self-efficacy showed a stepwise correlation with the FOIS score. More attention should be paid to the provision of care and coaching focused on defusing negative emotional experiences in each stage of dysphagia and self-efficacy. Our previous report showed the stepwise correlation between the HRQoL and the FOIS score [31]. Therefore, it may enhance the patient’s confidence in their swallowing abilities.

### 4.4. Intervention for Cancer Patients Suffering from Swallowing Dysfunction

Oral care involving rehabilitation of dysphagia and oral function, whether self-performed or performed by dentists and dental hygienists, is fundamental for preventing some adverse events (postoperative pneumonia, chemoradiotherapy-induced oral mucositis, taste disturbance, infection of the oral cavity, and swallowing disorders) [41]. Dysphagia is widely recognized as a common and debilitating side-effect of HNC and its treatment; however, minimal attention has been given to dysphagia in other cancer populations [3]. Several methods of rehabilitation for dysphagia in HNC were highlighted in a review [42]. Most of the rehabilitation of HNC patients with dysphagia has focused on the pathophysiology of dysphagia, including objective assessment (videoendoscopic evaluation of swallowing, videofluoroscopic examination of swallowing, dysphagia severity scale, and the FOIS) [43]. However, a psychosocial approach should be emphasized for the cancer patients with dysphagia. The food judged by the medical staff as edible for cancer patients is not the same food that the patients can eat or want to eat. In a prospective cohort study of 100 patients with either transient ischemic attack or ischemic stroke, Brouwer et al. found that a baseline of self-efficacy, as determined by patient’s responses on the questionnaires, was the strongest predictor of a patient’s intention to adopt a healthy diet (95% CI, 0.23–0.75) [24]. Thus, based on the aforementioned studies, it may yield benefit for hospitals’ neurosurgery and neurology departments to coordinate long-term stroke coaching programs and assess patients’ behavioral patterns to increase the probability of patients adhering to healthy lifestyles. On the other hand, in HNC patients, Roganie et al. reviewed only 15 (8 randomized) behavior change technique (BCT) reports, and the more frequent ineffective interventions used by BCT were practical social support, behavioral practice, self-monitoring of behavior, and credible source, for example, a skilled clinician delivering the intervention. As a result, swallowing interventions feature multiple components that may potentially impact outcomes [44]. These BCTs may improve the discrepancy of objective and subjective evaluations of dysphagia.

This study had some limitations. First, our study design was cross-sectional, and thus, a causal relationship between dysphagia and self-efficacy is unclear. In particular, the stepwise tendency between self-efficacy and FOIS was shown, but it was evaluated at a specific instance. Thus, a future study is required to verify the stepwise relationship between self-efficacy and FOIS using a longitudinal study design. Second, the participants who visited the oral care center had good oral health-related knowledge and attitudes; therefore, it is likely that they represented a higher oral health related-self-efficacy. Therefore, selection bias might exist. Although we consider that observational studies cannot avoid such selection bias, we believe that our findings provide important suggestions for performing randomized controlled trials in the future, which would reveal the actual impact of self-efficacy. Third, the SEAC lacks test–retest reliability and construct validity in previous studies. Therefore, our result showing the relationship between FOIS and SEAC may have a possible shortcoming of reproducibility.

## 5. Conclusions

Generally, self-efficacy played an important role in dysphagia and may affect the severity of dysphagia. We medical practitioners need to bridge the gap between the foods that cancer patients can subjectively eat and the foods that cancer patients can objectively eat.

## Figures and Tables

**Table 1:** The Functional Oral Intake Scale (FOIS).

Score	Performance	Implication	Deficit
1	Aspirates saliva/Tubedependent	Nothing by mouth	Profound
2	Tube dependent	Nothing by mouth/Minimal trials	Profound
3	Tube dependent	Full trials by mouth	Severe
4	Total oral	Single texture trials	Moderate
5	Total oral	Multiple texture trials	Mild
6	Total oral	By mouth/restrictions	Minimal
7	Regular diet	By mouth/No restrictions	None

**Table 2:** Participants’ characteristics (*n* = 203).

Variable	Percentage (*n*)	Median (Range)
Age		71 (34–93)
Gender		
Male	63.5 (129)	
Female	36.5 (74)	
Body Mass Index		21.7 (19.7–38.7)
Alcohol consumption per week		0 (0–7)
Brinkman Index		0 (0–4480)
Number of co-residents		2 (0–7)
Employed (yes)	35.5 (72)	
Primary tumor site		
Stomach	13.8 (28)	
Colorectal	12.3 (25)	
Liver	5.4 (11)	
Lung	32.0 (65)	
Prostate	10.3 (21)	
Breast	8.4 (17)	
Head/neck	17.7 (36)	
Cancer stage		
I	38.4 (78)	
II	19.2 (39)	
III	20.7 (42)	
IV	21.7 (44)	
Treatment type		
Surgery	59.6 (121)	
Radiotherapy	2.5 (5)	
Chemotherapy	8.4 (17)	
Surgery plus radiotherapy	3.0 (6)	
Surgery plus chemotherapy	14.8 (30)	
Chemotherapy plus radiotherapy	4.9 (10)	
Surgery plus chemotherapy plus radiotherapy	6.9 (14)	
Number of months since last treatment		0 (0–297)
Eastern Cooperative Oncology Group Performance Status		
0	59.6 (121)	
1	23.2 (47)	
2	5.9 (12)	
3	10.8 (22)	
4	0.5 (1)	
Number of teeth		22 (0–32)
Dentures		
Upper		
None	63.5 (129)	
Partial	20.2 (41)	
Full	16.3 (33)	
Lower		
None	65.5 (133)	
Partial	18.7 (38)	
Full	15.8 (32)	
Brushing times per day		
0	5.9 (12)	
1	23.6 (48)	
2	34.5 (70)	
3 or more	36.0 (73)	
Family dentist		
Yes	69.5 (141)	
No	30.5 (62)	
Dental visit(s) since last year		
Yes	41.1 (84)	
No	58.6 (119)	
Functional Oral Intake Scale		
1	3.4 (7)	
2	0 (0)	
3	0.5 (1)	
4	3.4 (7)	
5	12.8 (26)	
6	23.2 (47)	
7	56.7 (115)	
Functional Oral Intake Scale Category 1		
High FOIS	43.3 (88)	
Low FOIS	56.7 (115)	
Category 2		
Good FOIS	79.8(162)	
Moderate FOIS	35 (17.2)	
Poor FOIS	3 (6)	
Self-efficacy Scale for Advanced Cancer		
Affect regulation self-efficacy		58.3 (8–100)
Symptom coping self-efficacy		51.7 (2–100)
Activities of daily life self-efficacy		63.3 (2–100)
Total Score		1050 (70–1800)
Oral Health-related Self-Efficacy Scale for Patients with Cancer		
Brushing Habits Self-efficacy		8 (3–12)
Oral Function Self-efficacy		11 (5–16)
Dental visit Self-efficacy		8 (3–12)
Symptom Coping Self-efficacy		9 (3–12)
Adverse Effects Self-efficacy		10 (4–16)
Total Score		45 (30–68)

**Table 3:** The comparison of the high versus low FOIS analysis of each item.

Variable	Percentage (*n*) or Median (Range)		*p*-Value
	High FOIS (*n* = 88)	Low FOIS (*n* = 115)	
Age	72 (34–93)	69 (36–91)	<0.01 *
Gender			
Male	66 (75)	63 (54.8)	<0.01 *
Female	22 (25)	52 (45.2)	
Body Mass Index	21.3 (15.2–28.8)	22.4 (19.3–38.7)	0.04 *
Alcohol consumption per week	0 (0–7)	0 (0–7)	0.45
Brinkman Index	160 (0–4160)	0 (0–4480)	<0.01 *
Number of co-residents	2 (0–7)	1 (0–7)	0.08
Employed			
Yes	30 (34.1)	42 (36.5)	0.77
No	58 (65.9)	73 (63.5)	
Primary tumor site			
Stomach	18 (20.5)	10 (8.7)	0.02
Colorectal	13 (14.8)	12 (10.4)	0.35
Liver	4 (4.5)	7 (6.1)	0.63
Lung	18 (20.5)	47 (40.9)	<0.01 *
Prostate	9 (10.2)	12 (10.4)	0.96
Breast	1 (1.1)	16 (13.9)	<0.01 *
Head/neck	25 (28.4)	11 (9.6)	<0.01 *
Cancer stage			
I	20 (22.7)	58 (50.4)	<0.01 *
II	16 (18.2)	23 (20)	
III	21 (23.9)	21 (18.3)	
IV	31 (35.2)	13 (11.3)	
Treatment type			
Surgery	38 (43.2)	83 (72.2)	<0.01 *
Radiotherapy	1 (1.1)	4 (3.5)	0.29
Chemotherapy	10 (11.4)	7 (6.1)	0.18
Surgery plus radiotherapy	4 (4.5)	2 (1.7)	0.24
Surgery plus chemotherapy	18 (20.5)	12 (10.4)	0.05 *
Chemotherapy plus radiotherapy	6 (6.8)	4 (3.5)	0.28
Surgery plus chemotherapy plus radiotherapy	11 (12.5)	3 (2.6)	<0.01 *
Number of months since last treatment	1 (0–297)	0 (0–216)	0.06
Eastern Cooperative Oncology Group Performance Status			
0	29 (33)	92 (80)	<0.01 *
1	31 (35.2)	16 (13.9)	
2	9 (10.2)	3 (2.6)	
3	18 (20.5)	4 (3.5)	
4	1 (1.1)	0 (0)	
Number of teeth	15 (0–32)	25 (0–32)	<0.01 *
Brushing times per day			
0	10 (11.4)	2 (1.7)	<0.01 *
1	27 (30.7)	21 (18.3)	
2	25 (28.4)	45 (39.1)	
3 or more	26 (29.5)	47 (40.9)	
Family dentist			
Yes	46 (52.3)	95 (82.6)	<0.01 *
No	42 (47.7)	20 (17.4)	
Dental visit(s) since last year			
Yes	25 (28.4)	59 (51.3)	<0.01 *
No	63 (71.6)	56 (48.7)	
Self-efficacy Scale for Advanced Cancer			
Affect regulation self-efficacy	50.8 (8–97)	61.7 (13–100)	<0.01 *
Symptom coping self-efficacy	50 (2–90)	53.3 (7–100)	0.07
Activities of daily life self-efficacy	52.5 (2–93)	68.3 (17–100)	<0.01 *
Total Score	915 (70–1640)	1100 (280–1800)	<0.01 *
Oral Health-related Self-Efficacy Scale for Patients with Cancer			
Brushing Habits Self-efficacy	8 (3–12)	8 (3–12)	0.31
Oral Function Self-efficacy	10 (5–16)	11 (5–16)	<0.01 *
Dental visit Self-efficacy	8 (3–12)	9 (3–12)	0.31
Symptom Coping Self-efficacy	9 (6–12)	9 (3–12)	0.96
Adverse Effects Self-efficacy	10 (4–16)	10 (4–16)	0.53
Total Score	44 (33–64)	46 (30–68)	0.05 *

* = *p* < 0.05.

**Table 4:** Correlation between related factor(s) and High or Low FOIS by multivariate analysis.

Variable	Regression Coefficient	Wald	Significance Probability	Odds Ratio (95% Confidence Interval)	EXP(B)	
					Lower Limit	Upper Limit
Number of co-residents	−0.36	6.54	<0.01 *	0.70	0.53	0.92
Employed	−0.94	2.79	0.10	0.39	0.13	1.18
Stomach cancer	1.62	3.87	0.05 *	5.05	1.01	25.33
Colorectal cancer	3.76	13.37	<0.01 *	42.74	5.71	319.92
Liver cancer	3.85	8.95	<0.01 *	46.95	3.77	584.4
Lung cancer	3.62	20.0	<0.01 *	37.13	7.62	181.1
Prostate cancer	2.42	6.27	<0.01 *	11.2	1.69	74.29
Breast cancer	5.81	8.17	<0.01 *	334.15	6.21	17,969.17
Cancer stage	−0.52	4.98	0.03 *	0.60	0.38	0.94
Radiotherapy	3.48	2.38	0.12	32.38	0.39	2674.8
Surgery plus chemotherapy	−1.19	2.82	0.09	0.30	0.08	1.22
Eastern Cooperative Oncology Group Performance Status	−0.65	5.56	0.02 *	0.52	0.31	0.90
Number of teeth	0.15	25.2	<0.01 *	1.16	1.09	1.23
Family dentist	1.88	10.21	<0.01 *	6.55	2.07	20.75
Activities of daily life self-efficacy	0.04	4.42	0.04 *	1.04	1.00	1.07
Symptom Coping Self-efficacy	−0.49	7.04	<0.01 *	0.61	0.42	0.88
Constant	2.66	0.88	0.35	14.354		

* = *p* < 0.05, EXP(B): exponentiation of the B coefficient. The factors of age and gender were adjusted.
